# Improvement of Physical Performance Following a 6 Week Change-of-Direction Training Program in Elite Youth Soccer Players of Different Maturity Levels

**DOI:** 10.3389/fphys.2021.668437

**Published:** 2021-05-24

**Authors:** Dorsaf Sariati, Raouf Hammami, Hassane Zouhal, Cain C. T. Clark, Ammar Nebigh, Mokhtar Chtara, Sabri Gaied Chortane, Anthony C. Hackney, Nizar Souissi, Urs Granacher, Omar Ben Ounis

**Affiliations:** ^1^Tunisian Research Laboratory, Sport Performance Optimization, National Center of Medicine and Science in Sports (CNMSS), Tunis, Tunisia; ^2^Higher Institute of Sport and Physical Education of Ksar-Said, University of La Manouba, Manouba, Tunisia; ^3^Research Laboratory: “Education, Motricity, Sports and Health” (UR 15JS01), Higher Institute of Sport and Physical Education of Sfax, University of Sfax, Sfax, Tunisia; ^4^University of Rennes, M2S (Laboratoire Mouvement, Sport, Santé), Rennes, France; ^5^Centre for Intelligent Healthcare, Coventry University, Coventry, United Kingdom; ^6^Department of Exercise and Sport Science, University of North Carolina at Chapel Hill, Chapel Hill, NC, United States; ^7^Physical Activity, Sport, and Health, UR18JS01, National Observatory of Sport, Tunis, Tunisia; ^8^Division of Training and Movement Sciences, University of Potsdam, Potsdam, Germany

**Keywords:** youth soccer, peak height velocity, change of direction speed, training adaptation, football

## Abstract

**Background:** Change-of-direction (CoD) is a necessary physical ability of a field sport and may vary in youth players according to their maturation status.

**Objectives:** The aim of this study is: to compare the effectiveness of a 6-week CoD training intervention on dynamic balance (CS-YBT), horizontal jump (5JT), speed (10 and 30-m linear sprint times), CoD with (15 m-CoD + B) and without (15 m-CoD) the ball, in youth male soccer players at different levels of maturity [pre- and post-peak height velocity (PHV)].

**Materials and Methods:** Thirty elite male youth soccer players aged 10–17 years from the Tunisian first division participated in this study. The players were divided into pre- (G1, *n* = 15) and post-PHV (G2, *n* = 15) groups. Both groups completed a similar 6-week training program with two sessions per week of four CoD exercises. All players completed the following tests before and after intervention: CS-YBT; 5 JT; 10, 30, and 15 m-CoD; and 15 m-CoD + B, and data were analyzed using ANCOVA.

**Results:** All 30 players completed the study according to the study design and methodology. Adherence rate was 100% across all groups, and no training or test-related injuries were reported. Pre-PHV and post-PHV groups showed significant amelioration post-intervention for all dependent variables (after test > before test; *p* < 0.01, *d* = 0.09–1.51). ANOVA revealed a significant group × time interaction only for CS-YBT (*F* = 4.45; *p* < 0.04; η^2^ = 0.14), 5JT (*F* = 6.39; *p* < 0.02; η^2^ = 0.18), and 15 m-CoD (*F* = 7.88; *p* < 0.01; η^2^ = 0.22). CS-YBT, 5JT, and 15 m-CoD improved significantly in the post-PHV group (+ 4.56%, effect size = 1.51; + 4.51%, effect size = 1.05; and -3.08%, effect size = 0.51, respectively), more than the pre-PHV group (+ 2.77%, effect size = 0.85; + 2.91%, effect size = 0.54; and -1.56%, effect size = 0.20, respectively).

**Conclusion:** The CoD training program improved balance, horizontal jump, and CoD without the ball in male preadolescent and adolescent soccer players, and this improvement was greater in the post-PHV players. The maturity status of the athletes should be considered when programming CoD training for soccer players.

## Introduction

Change-of-direction (CoD) is a necessary physical ability of a field sport athlete (Lloyd et al., 2013; Sattler et al., 2015; Hammami et al., 2018). This is due to the inherent design of field-based team sports (i.e., soccer, handball, basketball, and lacrosse), which places great emphasis on the ability of the athlete to run quickly and change directions during a game (Sekulic et al., 2017). Despite the significance of CoD for sports performance, it has neither been a prominent factor in the long-term athlete development of athletic training programs nor well studied in research (Lloyd et al., 2015). Lloyd et al. (2015), in their recent review studying the effects of growth, maturation, and training on CoD in a safe and effective manner, showed that applying an optimal CoD training stimulus for the duration of athlete development is crucial for effective programming and improving CoD athletic performance in youth.

There is a lack of research in the pediatric athletic population, pertaining to the determinants of CoD performances in prepubertal and early pubertal athletes, while the recent Youth Physical Development (YPD) models (Lloyd et al., 2013; Granacher et al., 2016) accentuated the need for a structured and logical approach to developing different types of CoD during childhood and adolescence. In this context, previous cross-sectional studies have examined the interaction between maturation and CoD development in both pre-peak height velocity (PHV) (Hammami et al., 2017; Negra et al., 2017) and post-PHV handball players (Hammami et al., 2018). Accordingly, Hammami et al. (2018) indicated a stronger association between conditioning abilities (i.e., jumping and sprinting) and CoD in early pubescent handball players of advanced maturity status (i.e., post-PHV players). These authors reinforce the need for differential strength and conditioning programs aimed at improving the CoD of young athletes who differ in maturity status. While there is ample evidence on the CoD/strength-and-power relationship in youth, less is known regarding the effects of CoD training on particular measures of physical fitness with respect to maturation. Chaalali et al. (2016) demonstrated that elite young male soccer players’ physical performances can be significantly and specifically improved by either plyometrics or CoD or repeated sprint ability (RSA) training over short-term, in-season, training. In contrast, Padrón-Cabo et al. (2020) showed that coordination training with an agility ladder does not seem to be effective in improving physical fitness (i.e., 10-m sprint, 20-m sprint, dribbling speed test, and agility test) or dribbling (slalom dribbling test). Consequently, the purpose of the present study was to examine the effects of 6-week pre-season CoD training program on dynamic balance, horizontal jump (5 JT), speed (10- and 30-m linear sprint times), and CoD with and without the ball (15 m-CoD + B and 15 m-CoD tests, respectively), in male soccer players at different maturity statuses (pre- and post-PHV).

Concordant with earlier cross-sectional (Hammami et al., 2018) and longitudinal (Zemkova and Hamar, 2010; Asadi et al., 2017) investigations, we hypothesized that the more mature players would respond more favorably to CoD training.

## Materials and Methods

### Participants

Thirty young male soccer players (age: from 10 to 17 years) participated in the study (see Table 1). The players volunteered for random assignment to either CoD training program in both pre-PHV (*n* = 15) and post-PHV (*n* = 15) groups. Afterward, biological maturity was evaluated, non-invasively, by incorporating measures of chronological age and body height into a regression equation to subsequently predict biological age from PHV (Moore et al., 2015). The equation has previously been validated for boys and presents standard error of estimate reported as 0.542 years (Moore et al., 2015). By this equation, all players were classified as pre- or post-PHV, respectively. All participants were free from lower-limb musculoskeletal injuries, physically active, and participated regularly in soccer training. All participating players performed systematic soccer practice in the first division of the Tunisian national soccer league for 3–7 years. The players exercised on average five times per week with each session lasting ∼90 min and one match played during the weekend. Players were not undertaking any additional training other than the team soccer training.

**TABLE 1:** Design of the training program for both pre- and post-PHV soccer players.

**CoD drills**	**Week 1**	**Week 2**	**Week 3**	**Week 4**	**Week 5**	**Week 6**
Exercise 1	1 × 4	2 × 5	2 × 6	2 × 7	1 × 5	3 × 6
Exercise 2	1 × 4	2 × 5	2 × 6	2 × 7	1 × 5	3 × 6
Exercise 3	1 × 4	2 × 5	2 × 6	2 × 7	1 × 5	3 × 6
Exercise 4	1 × 4	2 × 5	2 × 6	2 × 7	1 × 5	3 × 6

*Set × repetition; example, (1 × 4) means that participants had to perform one set with four repetitions.*

Before participation in this study, the participants were given a letter that included written information about the study and a request for consent from the parents to allow their children to participate in this study. Legal representatives and players provided informed consent after a thorough explanation of the objectives and scope of this project, the procedures, risks, and benefits of the study. This study was conducted according to the latest version of the Declaration of Helsinki, and the protocol was fully approved by the Local Ethics Committee of the National Centre of Medicine and Science of Sports of Tunis (CNMSS-LR09SEP01) before the commencement of the assessments.

Players’ height and body mass were measured using a wall-mounted stadiometer and electronic scale, respectively. Body mass index (BMI) was calculated as mass/height squared (kg/m^2^). Two skinfold thicknesses (triceps and sub-scapular) were measured, in triplicate, by the same trained investigator. Measurements were made on the right-hand side of the body using a Harpenden caliper (Baty International, West Sussex, England). Body fat percentage was estimated using the equations of Slaughter et al. (1988) for boys.

### Sample Size

A minimum sample size of 30 was determined from an *a priori* statistical power analysis using G^∗^Power (Version 3.1, University of Dusseldorf, Germany) (Faul et al., 2009). The power analysis was computed with an assumed power at 0.90, an alpha level of 0.01, and a small effect size of 0.38 for our primary outcome, 10-m sprint time (Hammami et al., 2016).

### Procedures

All procedures were carried out during the first half of the competitive season. Before the commencement of the study, and prior to the initiation of baseline testing, all players completed a 2-week orientation period with three sessions per week to become familiar with the general environment, the applied physical fitness tests, and CoD exercises. A repeated-measures study design with pre–post tests and two experimental groups (i.e., pre- and post-PHV) was applied. Prior to testing, participants accomplished a standardized warm up consisting of low-intensity jogging, CoD, and balance and jumping exercises together with dynamic lower-limb stretching. Performance testing was conducted at the national team club “Ligue 1, Tunisia” pre–post the 6-week CoD training period and included the assessment of lower limb dynamic balance (CS-YBT), horizontal jump (5JT), CoD with (15 m-CoD + B) and without (15 m-CoD) the ball, and linear sprint performances (times over 10 and 30 m). The test sequence was randomized. Within 1 week, all tests were repeatedly performed and intra-class correlation coefficients (ICC), jointly with coefficients of variation (%CV), were assessed. Similar to other previously published training studies (McGawley and Andersson, 2013; Hammami et al., 2016), a true control group could not be included as the two experimental groups were national-level elite athletes and there were no comparable athletes available that would provide similar baseline values. Performance testing was initiated after a standardized 15-min warm-up program, including submaximal intensity running bouts; dynamic stretching; low-intensity forward, sideways, and backward running bouts; several accelerations; and vertical jumps. Intensity was progressively increased during the warm-up.

### Dynamic Balance

Stability was assessed by the Y-Balance Test according to a previously described protocol (Hammami et al., 2017; Sariati et al., 2020), which has been shown to be reliable in both pre-PHV [ICC = 0.92 and standard error of mean (SEM) = 2.54] (Hammami et al., 2017) and post-PHV soccer players (ICC = 0.91, SEM = 0.49) (Sariati et al., 2020). For this purpose, players stood on the dominant leg, with the most distal aspect of their big toe on the center of the grid.

Thereafter, they were asked to reach the maximal distance in the anterior (A), postero-medial (PM), and postero-lateral (PL) directions, while maintaining their single-limb stance (Fusco et al., 2020). The maximal reached distance was measured with a measuring tape as the most distal point reached by the free limb. The trial was discarded and repeated if the player failed to maintain a unilateral stance, touched down with the reaching foot, or failed to return the reaching foot to the starting position. A composite score [CS-YBT (%)] was calculated and considered as the dependent variable using the following formula: CS-YBT (%) = [(maximum anterior reach distance + maximum posteromedial reach distance + maximum posterolateral reach distance)/(leg length × 3)] × 100.

### Horizontal Jump

The five-jump test (5JT) was proposed to evaluate lower-limb horizontal jump in soccer players (Chamari et al., 2008). The testing protocol consists of five consecutive strides with joined feet position at the start and end of the jumps. From starting test, the participant was not approved to perform any back step with any foot; rather, he had to directly jump to the front with a leg of his choice. After the first four strides, i.e., alternating left and right feet for two times each, he had to perform the last stride and end the test again with joined feet. The test was performed again if the player fell back on completion of the last stride. Jumping performance was measured, in centimeters, with a tape measure from the front edge of the player’s feet at the starting position to the rear edge of the feet at the final position. The strength specialist assessing the landing had to focus on the last stride of the player in order to exactly determine the last foot placement on the grass, as the players could not always stay on their feet on landing. The starting position was set on a fixed point (Chamari et al., 2008). Previous test reliability conducted with youth elite soccer players was considered high with an ICC = 0.91 and SEM = 0.30, respectively (Sariati et al., 2020).

### Change of Direction With (15 m-CoD + B) and Without (15 m-CoD) the Ball

**FIGURE 1:**
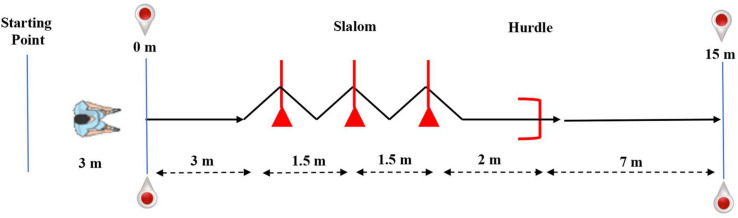
A Schematic representation of the change-of-direction (CoD) test.

For the 15 m-CoD test (Figure 1), players departed running 3 m behind the initial set of gates.

Players performed 3 m of straight running, entered a 3-m slalom section marked by three aligned sticks (1.6 m of height) placed 1.5 m apart, and then cleared a 0.5-m hurdle placed 2 m beyond the third stick. Finally, players ran 7 m to break the second set of photocell gates, which stopped the timer (Mujika et al., 2009). An excellent test–retest reliability has been reported for the 15 m-CoD run test, with an ICC value of 0.93 (0.86, 0.97) (Chaalali et al., 2016).

The 15 m-CoD + B run test (Figure 1) is alike to the previously described 15 m-CoD shuttle run; however, players needed to dribble a ball while executing the test. Next, the slalom section of the test, the ball was kicked under the hurdle while the player cleared it. The participant then kicked the ball toward either of two small goals placed diagonally 7 m on the left and the right sides of the hurdle and ended with 7 m of straight sprint (Mujika et al., 2009). Previous test–retest reliability scores for the 15 m-CoD + B have been shown to be reliable in a similar pediatric population (ICC = 0.87, SEM = 0.04) (Chaalali et al., 2016). Players performed two trials of each test (3-min rest between trials), and the best performance was used for further analysis.

### Linear Sprint Speed

Sprint performance was calculated using a stationary 10-m sprint and 30-m maximal speed test. The 10-m sprint comprised sprinting 10 m, as fast as possible. For all players, start stance was consistent. The 30-m maximal speed sprint comprised also sprinting 30 m as quick as possible from a moving start line. Participants were located 20 cm behind the starting line position and were instructed to run as fast as possible along the allocated distance. Sprint time was automatically noted using photocell gates (Brower Timing Systems, Salt Lake City, UT, United States; accuracy of 0.01 s) placed 0.4 m above the ground. Players performed two trials, with at least 5 min of rest between each trial, and the best trial was recorded for analysis. Previous test–retest reliability has demonstrated a high score in youth soccer players (Hammami et al., 2016; Makhlouf et al., 2018).

### Change-of-Direction Training Program

All players trained five times per week (i.e., ∼90 min per session) with one match played during the weekend over the entire training period. Players of the two groups participated in a 6-week CoD training program, which was performed twice per week (see Table 1). Training volume was similar between groups during the study. Each training session endured 20–25 min and included four CoD exercises.

**FIGURE 2:**
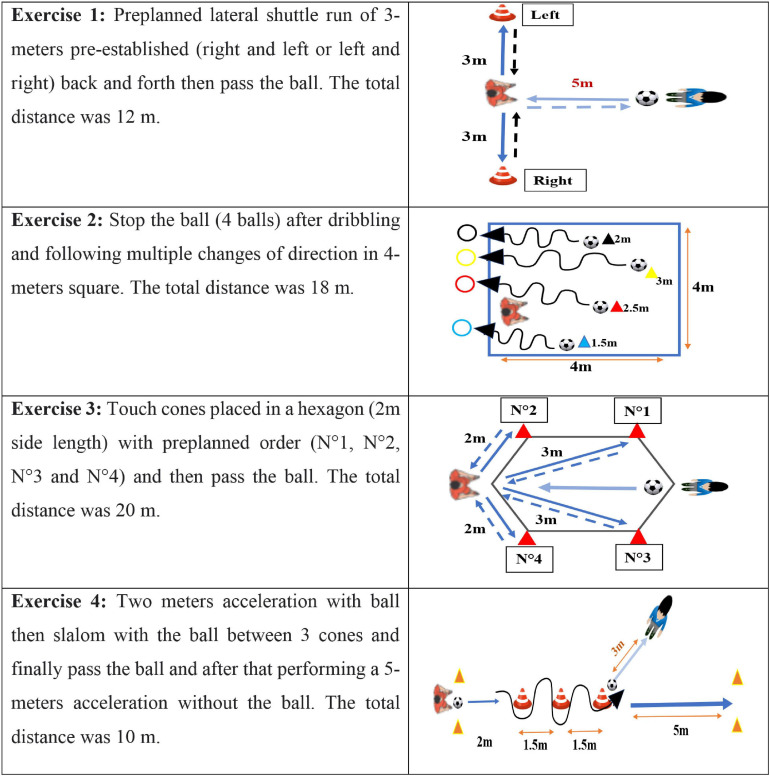
Description of the various change-of-direction speed training exercises.

A recovery time of approximately 50 s was allowed between trials and 2–3 min between sets. The CoD training program involved a number of CoD actions usually used by players seeking to improve CoD performance (see Figure 2). All CoD sessions were supervised by certified strength and conditioning specialists. During all training sessions, the coaches provided verbal encouragements.

### Statistical Analyses

Descriptive data were reported as means ± standard deviations (SD). Normal distribution of data was confirmed using the Shapiro–Wilk test, and baseline between-group differences were computed using independent samples *t*-tests. Subsequently, a 2 (group: pre-PHV and post-PHV) × 2 (test: pre and post) analysis of variance with repeated measures was used to identify the effects of the CoD training program on performance at different levels of maturity. Furthermore, group-specific repeated-measures analyses of variance (ANOVA) (time: before and after) were applied to evaluate within-group before-to-after performance changes. The effect size was calculated for all ANOVAs using partial eta-squared. The values of 0.01, 0.06, and 0.15 were considered as small, medium, and large cut-off points, respectively (Cohen, 1988). Effect sizes (ESs) were calculated for all paired comparisons and judged according to the following scale: ≤0.2, trivial; >0.2–0.6, small; >0.6–1.2, moderate; >1.2–2.0, large; and >2.0, very large (Hopkins et al., 2009). Test/retest reliability was assessed with Cronbach’s model intraclass correlation coefficient (ICC) and coefficient of variation (CV). We considered an ICC over 0.90 as high, between 0.80 and 0.90 as moderate, and below 0.80 as low (Vincent, 1995), and CV values were considered acceptable if <10% (Cormack et al., 2008). For each ICC, the 95% confidence interval (CI) was calculated to take the sampling distribution into account. Statistical analyses were performed using SPSS (SPSS Inc., Chicago, IL, version. 20.0), and significance was accepted, *a priori*, at *p* < 0.05.

## Results

All players from pre-PHV and post-PHV groups completed the study according to the prescribed protocol. Participants attended all training sessions, and no training- or test-related injuries were reported. Reliability measures (ICC) for the assessed tests ranged from 0.90 to 0.96, while CV ranged from 1.27 to 3.98% (Table 2).

**TABLE 2:** Intraclass correlation coefficients (ICCs) for relative reliability and coefficients of variation for absolute reliability of the applied physical fitness tests.

**Measures**	**ICC**	**95% CI**	**% CV**
CS-YBT (%)	0.91	0.81–0.96	1.59
5JT (m)	0.91	0.80–0.96	3.67
Sprint 10-m (s)	0.96	0.91–0.98	1.45
Sprint 30-m (s)	0.95	0.90–0.98	1.27
15 m-CoD (s)	0.96	0.92–0.98	2.55
15 m-CoD + B (s)	0.90	0.80–0.95	3.98

*ICC, intraclass correlation coefficient; CI, confidence interval; CV, coefficient of variation (%); 5JT, five-jump test; CS-YBT, Y-Balance Ttest composite score; 15 m-CoD, change-of-direction without the ball; 15 m-CoD + B, change-of-direction with the ball.*

The analysis revealed a significant difference between groups for all anthropometric measurements, except BMI (*p* = 0.13). Accordingly, the post-PHV group was more advanced in age (*p* = 0.001; *d* = 18.40), maturity offset (*p* = 0.001; *d* = 12.20), height (*p* = 0.001; *d* = 2.21), and body mass (*p* = 0.001; *d* = 1.82) than the pre-PHV group. Inversely, the pre-PHV group was lower than the post-PHV in body fat (BF) (*p* = 0.02; *d* = 0.93) (Table 3).

**TABLE 3:** Participants’ anthropometric characteristics by player’s maturity status.

	**Pre-PHV (*n* = 15)**	**Post-PHV (*n* = 15)**	**Cohen’s *d***	***p*-value**
Age (years)	10.9 ± 0.4	17.4 ± 0.3	18.40	0.001
PHV (years)	–1.5 ± 0.3	3.2 ± 0.5	12.20	0.001
Height (cm)	164.9 ± 5.0	177.3 ± 6.5	2.21	0.001
BM (kg)	55.0 ± 6.4	67.0 ± 7.2	1.82	0.001
BMI (kg/m^2^)	20.2 ± 1.9	21.3 ± 1.9	058	0.13
BF (%)	9.9 ± 2.2	12.2 ± 2.7	0.93	0.02

*Data are means ± standard deviations (SD). BM, body mass; BF, body fat; BMI, body mass index, PHV, peak height velocity.*

The analyses indicated a significant main effect of time (*p* < 0.001; η^2^ = 0.53–0.79) for all dependent variables. Both groups showed significant improvements, after intervention (after test > before test; *p* < 0.01) (Table 4). In addition, ANOVA revealed a significant group × time interaction only for CS-YBT (*F* = 4.45; *p* < 0.04; η^2^ = 0.14), 5JT (*F* = 6.39; *p* < 0.02; η^2^ = 0.18), and 15 m-CoD (*F* = 7.88; *p* < 0.01; η^2^ = 0.22). CS-YBT, 5JT, and 15 m-CoD improved significantly in post-PHV to a greater extent (+ 4.56%, effect size = 1.51; + 4.51%, effect size = 1.05; and –3.08%, effect size = 0.51, respectively) than pre-PHV (+ 2.77%, effect size = 0.85; + 2.91%, effect size = 0.54; and –1.56%, effect size = 0.20, respectively) (Table 4).

**TABLE 4:** Effects of 6-week CoD training program on dynamic balance, horizontal jump, speed 10- and 30-m linear sprint times, and CoD with and without the ball (15 m-CoD and 15 m-CoD + B) in male soccer players at different maturity status (pre-PHV and post-PHV) (mean ± SD).

**Variables**	**Group**	**Before training**	**After training**	**Change%**	**Cohen’s *d***	**ANOVA *p*-value (η^2^)**		
						**Time**	**Group**	**Time × group**
CS-YBT (%)	Pre-PHV	76.28 ± 2.48	78.40 ± 1.61**	+ 2.77	0.85	0.001 (0.70)	0.001 (0.41)	**0.04** (0.14)
	Post-PHV	79.06 ± 2.39	82.66 ± 2.92**	+ 4.56	1.51			
5JT (m)	Pre-PHV	10.78 ± 0.58	11.09 ± 0.54**	+ 2.91	0.54	0.001 (0.74)	0.001 (0.73)	**0.02** (0.18)
	Post-PHV	12.35 ± 0.53	12.90 ± 0.52**	+ 4.51	1.05			
Sprint 10-m (s)	Pre-PHV	1.90 ± 0.05	1.88 ± 0.05**	–0.88	0.36	0.001 (0.78)	0.001 (0.71)	0.36 (0.03)
	Post-PHV	1.75 ± 0.05	1.73 ± 0.05**	–1.14	0.40			
Sprint 30-m (s)	Pre-PHV	4.24 ± 0.16	4.21 ± 0.16**	–0.66	0.18	0.001 (0.66)	0.001 (0.23)	0.37 (0.03)
	Post-PHV	4.08 ± 0.15	4.06 ± 0.14**	–0.54	0.15			
15 m-CoD (s)	Pre-PHV	3.29 ± 0.26	3.24 ± 0.27**	–1.56	0.20	0.001 (0.79)	0.001 (0.48)	**0.01** (0.22)
	Post-PHV	2.90 ± 0.17	2.81 ± 0.16**	-3.08	0.51			
15 m-CoD + B (s)	Pre-PHV	4.14 ± 0.36	4.11 ± 0.36**	–0.74	0.09	0.001 (0.53)	0.008 (0.43)	0.41 (0.02)
	Post-PHV	3.67 ± 0.17	3.63 ± 0.17**	–1.13	0.25			

**Significant difference between before and after training, p < 0.05. ES, effect size; 5JT, five-jump test; CS-YBT, composite score during the Y-Balance Test; 15 m-CoD, change-of-direction without the ball; 15 m-CoD + B, change-of-direction with the ball. Bold values represent statistically significant values and were therefore highlighted.

## Discussion

This study sought to examine the effects of a 6-week CoD training program on dynamic balance, horizontal jump, CoD with and without the ball, and sprint performance in youth male soccer players of different maturity status (pre-PHV and post-PHV). The within-group analyses revealed that the inclusion of the CoD training program increased almost all of the outcome measures in both pre-PHV and post-PHV groups, although larger effects were observed in dynamic balance, horizontal jump, and CoD without the ball for the post-PHV group (see Table 3). To the authors’ knowledge this is the first study to demonstrate the effectiveness of the CoD training program in improving balance, horizontal jump, CoD, and sprint performance in male preadolescent and adolescent soccer players and to specifically compare the influence of different maturity status on this outcome.

Focusing on the within-group analysis (comparison baseline–follow-up), after 6 weeks of CoD training program, the post-PHV group further enhanced their performance in the dynamic balance (CS-YBT), horizontal jump (5JT), and CoD (15-m shuttle run without the ball). The post-PHV young soccer players outperformed the pre-PHV group in measures of dynamic balance (CS-YBT). The principal mechanism of the training adaptations may be associated to the balance challenges related with CoD training.

The successful execution of the CoD exercise requires the ability to rapidly accelerate, decelerate, and change position from side-to-side. Indeed, in such demanding situations, the vestibular system must compensate and adjust as the CoD task requires a repeated shift in the center of gravity outside the base of support, which challenges bodily equilibrium (Hammami et al., 2017, 2021).

The ability to move the center of gravity outside the base of support and maintain good balance and stability is defined by metastability (Kibele et al., 2015) and is considered as a key component of athletic performance and soccer success. CoD training also has many advantages over a stationary balance training (Makhlouf et al., 2018). In fact, it is a dynamic, high-speed, explosive activity and therefore joining to the concept of training specificity in soccer. The importance of dynamic balance has been further argued in a 6-week agility training program that was conducted to a greater performance improvement (Zemkova and Hamar, 2010), while numerous authors have posited that improvement in balance performance or metastability may be correlated with change of direction or agility tasks (Mirkov et al., 2008; Hammami et al., 2017). The present study indicated that the CoD training program may have triggered acute mechanisms that contribute to a better dynamic balance performance in post-PHV soccer players. Since CoD imposes many perturbations during balance and stability, the ability to efficiently support static and dynamic balance (metastability) could positively improve athletic (i.e., soccer) performance (Jones et al., 2009).

There were time and group effects for the testing measures of horizontal jump (5JT), indicating general overall improvements in the post-PHV group. The specific performance changes might be partly attributed to physical influences from growth and maturation (Malina et al., 2004). Moreover, the prior soccer training of these elite players also plays a role; given that soccer involves sprinting, jumping, and CoD tasks (Stolen et al., 2005; Ramirez-Campillo et al., 2014), the lower legs would already be in a relatively highly trained mood (high muscle power performance) and may have been highly susceptible to this short bout of training for enhancing muscle power (i.e., horizontal jumping). Consequently, for pre-PHV soccer players, a longer duration or more frequent training program may be required to generate further training adaptations from muscle groups that undergo power activities on almost a daily basis.

The present results showed that the 15 m-CoD test was more improved in the post-PHV group. According to similar maturational studies (Zemkova and Hamar, 2010; Lloyd et al., 2013; Asadi et al., 2017; Hammami et al., 2018), it could be speculated that the high neural demand of rapidly changing directions required a stimulus that coincided with the natural adaptive response of the post-PHV participants, resulting in growth and maturation (Lloyd et al., 2013). Developmentally, post-PHV players experienced neuromuscular (e.g., improved motor unit recruitment and firing rate) and morphological changes (e.g., improved pennation angles) that facilitate force generation, in addition to cognitive maturation (Malina et al., 2004). Therefore, future studies investigating the effect of cognitive maturation on pre-planned performance development are warranted.

In addition, major morphological and neural changes occur with growth and maturation (Malina et al., 2004; Tonson et al., 2008). These asynchronously changing parameters in youth play an important role in the ability to adapt to a CoD-specific training stimulus (Eisenmann and Malina, 2003; Vanttinen et al., 2011). Hence, knowledge of when to apply an appropriate CoD training stimulus during long-term athlete development is essential for effective programming and improving athletic related-performance.

Moreover, since the CoD training program did not involve more advanced skill training with a soccer ball, it might be expected that soccer-specific coordination skills (i.e., 15 m-CoD + B) would not be positively affected by CoD training, although this assertion should empirically test. Finally, as maturation has been shown to contribute to the trainability of CoD ability (Lloyd et al., 2013), the rate at which these individuals are experiencing maturation may, in part, explain the variation in training response. Considering that responses to training stimuli can differ between these maturation groups (Lloyd et al., 2013), it would be beneficial to examine such effects in the future. In addition, although improvements in balance, horizontal jump, and CoD were evident within and between maturation groups, future studies should holistically examine the effects of the CoD training program in youth athletes accounting for changes, such as muscle action and muscle architecture, in addition to the effect on further physical components, such as lower-limb asymmetry, with consideration of injury prevention.

Although the present study presents a novel addition to the literature, which has, hitherto, not been reported, there are some limitations that warrant consideration. Firstly, we acknowledge the small sample size, which reduces our statistical power; however, this study merely presents a preliminary work, and not designed to be powered at the level of a randomized controlled trial, and provides a stepping stone for further work.

Secondly, the duration of the intervention may be regarded a limitation; indeed, only 6 weeks can be considered a relatively short duration, which may have precluded more marked changes in test variables. Nevertheless, these short periods are also dictated by training periodization over the course of a soccer season. In addition, we were not able to examine the underlying neuromuscular mechanisms responsible for the observed improvement in measures of physical fitness due to the lack of neurophysiological testing apparatus in the design of this study. Therefore, future studies are advised to include electrophysiological testing apparatus (e.g., electromyography), although researchers and practitioners are encouraged to consider the practical benefits with logistical demands of such testing.

Finally, the CoD test used in the present study contained different magnitudes of other physical components (e.g., hurdle jump) where anaerobic capacity is a critical factor in performance, making it difficult to discern whether changes in performance are due to increases in CoD ability or improvements in anaerobic capacity (Nimphius et al., 2018). Future research should consider the adoption of more ecological CoD tests.

## Conclusion

The present study is, to the authors’ knowledge, the first to examine the effects of a COD training program on balance, horizontal jump, and CoD performance in male youth soccer players of different maturation levels. Based on the extant literature (Zemkova and Hamar, 2010; Lloyd et al., 2013; Asadi et al., 2017) and the improvements found in the current study in balance, horizontal jump, and CoD performance in the post-PHV soccer players, a greater emphasis on prior CoD activities and training should be placed on younger athletes (pre-PHV soccer players) with less developed neuromuscular systems in order to improve efficacy of balance, power, and CoD training. The COD training program utilized in this study may help practitioners working with pre-adolescent and adolescent male soccer players to improve their training programs.

## Data Availability Statement

The raw data supporting the conclusions of this article will be made available by the authors, without undue reservation.

## Ethics Statement

The study was conducted according to the latest version of the Declaration of Helsinki and the protocol was fully approved by the Local Ethics Committee of the National Centre of Medicine and Science of Sports of Tunis (CNMSS) before the commencement of the assessments. Written informed consent to participate in this study was provided by the participants’ legal guardian/next of kin.

## Author Contributions

DS, OO, RH, NS, SC, AN, and HZ participated in the conception and design of the study. DS, OO, RH, MC, and AN were responsible for testing. DS, MC, CC, UG, SC, NS, and AH were responsible for data collection and statistical analysis. DS, RH, MC, CC, SC, AN, AH, UG, NS, HZ, and OO were responsible for the writing and finalization of the manuscript. All authors contributed to the manuscript and approved the submitted version.

## Conflict of Interest

The authors declare that the research was conducted in the absence of any commercial or financial relationships that could be construed as a potential conflict of interest. The reviewer BD declared a past co-authorship with the authors CC and UG to the handling editor.
